# Supplementary material for: Fertilizer and herbicide alter nectar and pollen quality with consequences for pollinator floral choices
Source: PeerJ. 2023 Jun 12;11:e15452. doi: 10.7717/peerj.15452 (PMC10269573; doi:10.7717/peerj.15452)
Supplement: Supplemental Information 1 [file peerj-11-15452-s001.docx]

Supplemental Material for **Fertilizer and herbicide alter nectar and pollen quality with consequences for pollinator floral choices**

*Pollen Amino Acids*

First, we weighed the subsamples (3-6mg each) into glass tubes with 1mL hydrochloric acid (HCL) and mixed them. For analysis of total amino acids, 1 mL of hydrolysis solution (6 N HCl, 0.1 % phenol, and 500 μM norleucine) was added to the pollen. Then we aerated the tubes with N_2_ gas for 1 minute. We capped the tubes and incubated them at 110°C for 24 hours. We then evaporated the samples and filtered them using a 50mL syringe (with a 0.22µm – 13mm filter) and a citrate buffer (pH 2.2) into a 1.5mL glass vial with a rubber seal for the high-performance liquid chromatography (HPLC) injection. We used a spectrum analyser to identify the peaks of the individual amino acids, and the area under the curve of the spectra corresponded to the quantity of individual amino acids. Each amino acid was measured separately with an ion exchange chromatograph (Biochrom 20 Plus Amino Acid analyser). A post-column ninhydrin reaction produced coloured derivatives, which was monitored via a UV detector. For the amino acid extractions, norleucine was used as the internal standard [1].

*Pollen Fatty Acids*

We quantified the fatty acids in the pollen of each of six plant species and four treatments and analysed two subsamples of each pollen sample (48 total samples) [2]. First, we dried the pollen samples overnight at 30°C in a drying oven. Then, we sifted the pollen through a pollen sieve and weighed two subsamples (5-10mg each) of each pollen type into 1.5mL vials. The pollen samples were mixed with 1mL hexane and kept at 60C and 300rpm in a dry block heating thermostat thermomixer (Thermomixer Compact, Eppendorf, Hamburg, Germany) for 24 hours. We pre-conditioned paired 3mL silica gel columns (Macherey Nagel, Düren, Germany) using 2mL of an ethyl acetate/hexane solvent (80/1) (Sigma Aldrich, Taufkirchen, Germany and Merck, Darmstadt, Germany), and transferred the sample to the column using 0.5mL of the same solvent with 20µL of an internal fatty acid standard (0.2mg/mL nonadecanoic acid in methanol, both Sigma Aldrich). Then we washed the vial with another 0.5mL of the solvent to ensure we captured all of the pollen. The next steps were to elute the triglycerides, cholesterol and diacylglycerols: (1) we added 5mL of isooctane/ethyl acetate (20/1) (both Sigma Aldrich), (2) we rinsed the vial twice with 0.5mL of isooctane/ethyl acetate (75/25) and poured on the column, and (3) we added 5mL of isooctane/ethyl acetate (75/25). Finally, we eluted the free fatty acids by adding 5mL of isooctane/ethyl acetate/acetic acid (75/25/2) to the column. In each step, we additionally rinsed the original vial with two times 0.5mL of the corresponding solvent to wash out all fatty acids. We transferred the two the free fatty acid fractions (free and bound) to a glass vial with dichloromethane/methanol (2/1) and added 20µL of trimethylsulfonium hydroxide (TMSH) as derivatization agent to receive fatty acid methyl esters (FAMEs) which can be analysed via gas chromatography/mass spectrometry (GCMS).

We used a GCMS (5975C inert XL MSD, Agilent Technologies, Santa Clara, USA) to generate mass spectra chromatograms, which we analysed to identify the molecular weights of individual fatty acids. Via automatic liquid sampler, 1µL of each sample was injected in splitless mode. We used Helium as carrier gas. The oven temperature was 60°C, held for 1 min before heating at a rate of 15°C/minute to 150°C, held for 10 min. Afterward, the temperature was increased at 10°C/minute to 320°C, held for 10 min. Electron ionization mass spectra were recorded from m/z 40 to 650. Ion source and transfer line temperature were constant at 250°C. We used a FAME Mix, C4-C24 (Supelco, Bellefonte, USA) and the NIST MS Search 2.0 library to identify fatty acids via mass spectra. MSD ChemStation F.01.00.1903 was used to manually integrate FAME peaks in chromatograms. The peaks of oleic acid and linolenic acid could not be separated and were integrated as one mixed peak. The integrated area of the internal standard (nonadecanoic acid) was used to calculate the concentrations of identified fatty acids. Total fat content was calculated by summing up amounts of all fatty acids.

*Nectar*

To extract sugars and amino acids, we added 100µL of absolute ethanol into the Eppendorf tubes with the nectar samples, and vortexed to mix. We then centrifuged the tubes twice for 5 min each time to ensure they were fully mixed. We then evaporated the ethanol in a DURAN® dehydrator overnight. The next day, we added 50µL of distilled water and repeated the vortexing and centrifuging steps. We then transferred a 48µL subsample into a ND10 glass vials with micro inserts from each nectar sample to analyse. The analysis of the nectar amino acids and sugars was carried out chromatographically by means of HPLC (Agilent Technologies 1260 Series; Agilent, Böblingen, Germany).

The analysis of the nectar amino acids and sugars was carried out chromatographically by means of HPLC (Agilent Technologies 1260 Series; Agilent, Böblingen, Germany). There for 8μL (AA determination) and 30μL (sugar determination) from each subsample was injected into the system. To determine the AA, the reversed-phase method was used with pre-column derivatization using OPA (all primary AA) and FMOC (secondary AA, here: proline) with an Extend-C18 column (Zorbax: 3.0 9 150 mm, 3.5μm; Agilent) + pre-column used. The AA derivates then were detected by Diode Array Detector (DAD; Agilent 1260 Infinity System, G4212B) [3–5]. We ran the reference standard (AA Standard Sigma Aldrich, HPLC grade; contains 17 AA: L-Alanine, L-Arginine, L-Aspartic acid, L-Cystine, L-Glutamic acid, Glycine, L-Histidine, L-Isoleucine, L-Leucine, L-Lysine, L-Methionine, L-Phenylalanine, L-Proline, L-Serine, L-Threonine, L-Tyrosine, L-Valine) in four concentrations.

The sugars, on the other hand, were determined isocratically using an NH2 column (Zorbax: 4.6 9 250 mm, 5μm; Agilent) + pre-column as described in Venjakob et al. [4]. The Elution ran with [(ACN/ME-OH)(78/22)] and was detected by an Refractive Index Detector (RID; Agilent 1260 Infinity, G1362 A) [4–6]. A Four Sugar Mix Standard (sucrose, glucose, fructose, maltose HPLC grade; Sigma-Aldrich) ran in five concentrations. For the purposes of our analysis, we found we needed to further dilute the nectar to quantify the sugars because they were too concentrated to compare with our standards. For this reason, after we quantified the amino acids, we performed a 1:3 dilution on the remaining sample.

*Species Based Models*

Species level variation among the plants surveyed explained most of the variation in pollen and nectar nutrition. Because plant species identity was not our primary interest, we evaluate these effects in the supplement.

Using plant species as the fixed effect and treatment and replicate number as random effects was a significantly better model for the pollen amino acids (having a significantly lower AICc) than using the treatment as the fixed effect and species are the random effect. With species as the fixed effect the marginal R^2^ of the model was 0.92, implying species level variation explains 92% of the variation in the total amino acid content of the pollen. Moreover, there were significant differences among the different plant species, with *C. vulgare*, *H. radicata*, and *P. tanacetifolia* having significantly higher total amino acid concentrations than *E. hirsutum*, *F. ulmaria*, and *P. lanceolata* (Fig. 1, Table S2).

Similarly, the best model (significantly lower AICc) for variation in pollen fatty acids had species as a fixed effect. The marginal R^2^ for the model with species as a fixed effect was 0.28, suggesting that species identity explained 28% of the variation in pollen fatty acids. The plant species also differed significantly from one another with *C. vulgare*, *P. tanacetifolia*, and *P. lanceolata* having significantly higher fatty acid concentrations than *E. hirsutum*, *F. ulmaria*, and *H. radicata* (Fig. 1, Table S2).

In terms of the nectar, there were significant differences between the plant species. *E. hirsutum* had the highest nectar sugar, followed by *O. vulgare*, which had a significantly higher nectar sugar content than *P. tanacetifolia* (Fig. 1). Plant species identity explained 90% of the variation in nectar sugar (R^2^ of 0.90, Table S2).

The R^2^ for the nectar amino acids model with species as a fixed effect was 0.95, suggesting that species identity explained most of the variation in nectar amino acids. Among the plant species, *O. vulgare* had the highest total nectar amino acid content, followed by *E. hirsutum*, which had a significantly higher nectar amino acid content than *P. tanacetifolia* (Fig. 1, Table S2).

**Supplemental References**

1. Vanderplanck M, Moerman R, Rasmont P, Lognay G, Wathelet B, Wattiez R, Michez D. 2014 How does pollen chemistry impact development and feeding behaviour of polylectic bees? PLOS ONE 9, e86209. (doi:10.1371/journal.pone.0086209)

2. Trinkl M, Kaluza BF, Wallace H, Heard TA, Keller A, Leonhardt SD. 2020 Floral species richness correlates with changes in the nutritional quality of larval diets in a stingless bee. Insects 11, 125. (doi:10.3390/insects11020125)

3. Henderson Jr J, Brookes A. 2010 Improved amino acid methods using Agilent ZORBAX Eclipse lus C18 Columns for a variety of Agilent LC instrumentation and separation goals. Agilent Technologies 5990–4547E, 1–16.

4. Venjakob C, Ruedenauer FA, Klein AM, Leonhardt SD. 2022 Variation in nectar quality across 34 grassland plant species. Plant Biology 24, 134–144. (doi:10.1111/PLB.13343)

5. Venjakob C, Leonhardt S, Klein AM. 2020 Inter-individual nectar chemistry changes of field scabious, Knautia arvensis. Insects 11. (doi:10.3390/INSECTS11020075)

6. Din-Norm-10758 : 1997-05. Analysis of honey – Determination of the content of saccharides fructose, glucose, saccharose, turanose and maltose – HPLC method. Berlin: Deutsches Institut für Normierung, 1997. 4 pp.

**Table S1.** Grams of pollen per flower in the four experimental treatments (C: control, F: fertilizer, H: herbicide, HF: combination) for six plant species. Dehisced pollen was collected from *P. lanceolata*, *C. vulgare*, and *H. radicata*, while anthers were collected from *F. ulmaria*, *E. hirsutum,* and *P. tanacetifolia*.

| Species | **C** | **F** | **H** | **HF** |
| --- | --- | --- | --- | --- |
| *Cirsium vulgare* | 0.132 | 0.122 | 0.16 | **0.277** |
| *Epilobium hirsutus* | 1.715 | 1.95 | 1.893 | **2.387** |
| *Filipendula ulmaria* | 0.887 | **2.235** | 1.629 | 1.348 |
| *Hypochaeris radicata* | 0.282 | 0.309 | 0.189 | **0.351** |
| *Phacelia tanacetifolia* | 0.753 | **0.909** | 0.568 | 0.813 |
| *Plantago lanceolata* | 0.741 | **1.128** | 0.413 | 0.979 |

**Table S2.** Results of GLMM models, significant contrasts shaded and bolded. We include the model structure (response and its transformation, fixed effects, random effects, and the contrasts). We also include the number of observations by group by the random effect for each model. The output from the model includes the effect size, p value (significance), and marginal and conditional R^2^. Marginal R^2^ expresses the percent of variation in the response explained by the fixed effects in the model, while the conditional R^2^ expresses the percent of the variation in the response explained by the entire model (fixed and random effects together). The effect size refers to the predicted magnitude of the relationship between the fixed effect and change in the response variable.

| **Response** | **Transformation** | **Contrast** | **Fixed effects** | **Random effects** | **Family** | **Observations** | **Groups** | **t value** | **Effect size** | **p value** | **R^2^m** | **R^2^c** |
| --- | --- | --- | --- | --- | --- | --- | --- | --- | --- | --- | --- | --- |
| Pollen Total Amino Acids | log | *C. vulgare -E. hirsutum* | Species | Replicate\|Trt | Gaussian | 73 | 4 | **-19.16** | **-1.26** | **< 0.001** | 0.92 | 0.92 |
|  |  | *C. vulgare -F. ulmaria* |  |  |  |  |  | **-19.76** | **-1.3** | **< 0.001** |  |  |
|  |  | *C. vulgare -H. radicata* |  |  |  |  |  | -0.81 | -0.05 | 0.42 |  |  |
|  |  | *C. vulgare – P. tanacetifolia* |  |  |  |  |  | -1.76 | -0.12 | 0.08 |  |  |
|  |  | *C. vulgare -P. lanceolata* |  |  |  |  |  | **-9.73** | **-0.64** | **< 0.001** |  |  |
| Pollen Total Fatty Acids | log | *C. vulgare -E. hirsutum* | Species | Replicate\|Trt | Gaussian | 48 | 4 | **-2.51** | **-0.60** | **0.01** | 0.28 | 0.5 |
|  |  | *C. vulgare -F. ulmaria* |  |  |  |  |  | **-3.63** | **-0.87** | **0.003** |  |  |
|  |  | *C. vulgare -H. radicata* |  |  |  |  |  | **-3.46** | **-0.83** | **0.005** |  |  |
|  |  | *C. vulgare – P. tanacetifolia* |  |  |  |  |  | -0.70 | -0.17 | 0.49 |  |  |
|  |  | *C. vulgare -P. lanceolata* |  |  |  |  |  | -0.31 | -0.07 | 0.76 |  |  |
| Nectar Sugar | log | *E. hirsutum – O. vulgare* | Species | Trt | Gaussian | 12 | 4 | **-5.57** | **-1.00** | **< 0.001** | 0.9 | 0.92 |
|  |  | *E. hirsutum – P. tanacetifolia* |  |  |  |  |  | **-11.41** | **-2.05** | **< 0.001** |  |  |
| Nectar Total Amino Acids |  | *E. hirsutum – O. vulgare* | Species | Trt | Gaussian | 12 | 4 | **14.05** | **1.23** | **< 0.001** | 0.95 | 0.99 |
|  |  | *E. hirsutum – P. tanacetifolia* |  |  |  |  |  | **-13.75** | **-1.20** | **< 0.001** |  |  |

**Table S3.** GLMM results for the visitation rates of different flower-visitor subgroups to pollen attributes including pollen to lipid ratio (P:L Ratio), here calculated as the total pollen amino acid concentration divided by the total pollen fatty acid concentration. We include the model structure (response, fixed effects, random effects, and the contrasts). We also include the number of observations by group (grouped by the random effect) for each model. The output from the model includes the effect size, p value (significance), and marginal and conditional R^2^. The effect size refers to the predicted magnitude of the relationship between the fixed effect and change in the response variable.

| **Response** | **transformation** | **fixed effects** | **random effects** | **family** | **observations** | **groups** | **t value** | **effect size** | **p value** | **R^2^m** | **R^2^c** |
| --- | --- | --- | --- | --- | --- | --- | --- | --- | --- | --- | --- |
| Total Visit Rate | log | **P:L Ratio** | Site | Gaussian | 231 | 8 Sites | **-2.47** | **-1.00E-03** | **0.01** | 0.17 | 0.19 |
|  |  | **Pollen Total AA** |  |  |  |  | **3.96** | **5.00E-04** | **0.00007** |  |  |
|  |  | Pollen Total FA |  |  |  |  | -1.86 | -8.00E-04 | 0.06 |  |  |
| Honeybee Visit Rate | log | P:L Ratio | Site | Gaussian | 231 | 8 Sites | -1.11 | -5.75E-05 | 0.27 | 0.01 | 0.07 |
|  |  | Pollen Total AA |  |  |  |  | 0.91 | 1.23E-05 | 0.36 |  |  |
|  |  | Pollen Total FA |  |  |  |  | -1.31 | -6.42E-05 | 0.19 |  |  |
| Syrphid Visit Rate | log | P:L Ratio | Site | Gaussian | 231 | 8 Sites | 1.03 | 1.63E-04 | 0.31 | 0.1 | 0.31 |
|  |  | Pollen Total AA |  |  |  |  | 0.57 | 2.35E-05 | 0.57 |  |  |
|  |  | Pollen Total FA |  |  |  |  | -0.14 | -2.09E-05 | 0.89 |  |  |
| Bumble bee Visit Rate | log | **P:L Ratio** | Site | Gaussian | 231 | 8 Sites | **-3.34** | **-1.18E-03** | **<0.001** | 0.15 | 0.15 |
|  |  | **Pollen Total AA** |  |  |  |  | **3.92** | **3.61E-04** | **<0.001** |  |  |
|  |  | **Pollen Total FA** |  |  |  |  | **-1.63** | **-5.41E-04** | **0.01** |  |  |
| All Bee Visit Rate | log | **P:L Ratio** | Site | Gaussian | 231 | 8 Sites | **-2.94** | **-1.12E-03** | **0.003** | 0.13 | 0.14 |
|  |  | **Pollen Total AA** |  |  |  |  | **3.78** | **3.75E-04** | **0.0002** |  |  |
|  |  | Pollen Total FA |  |  |  |  | -1.73 | -6.20E-04 | 0.08 |  |  |

**Figure S1.**
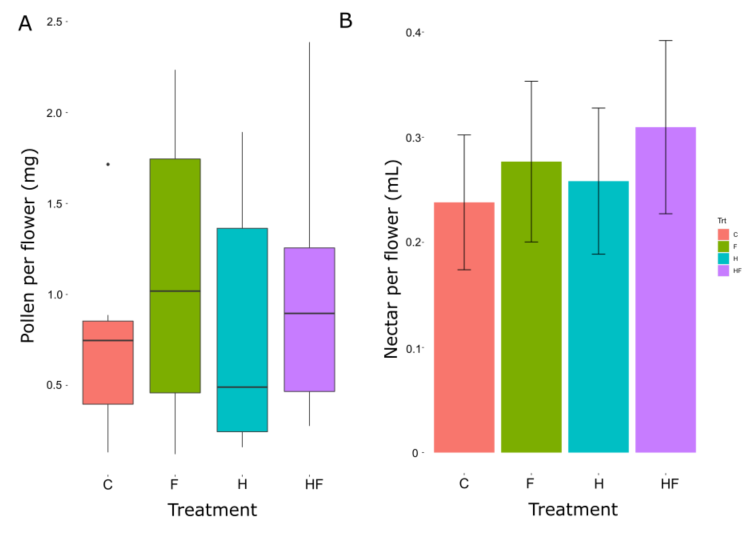


**Figure** **S1.** The amount of pollen (A) and nectar (B) collected per flower in the greenhouse experiment, separated by experimental treatment. Control = red, Fertilizer = green, Herbicide = blue, and Combination = purple. For the box-and-whisker plot (A), the box indicates the first and third quartiles, the whiskers indicate 1.5 * the interquartile range, and the black line indicates the median. Points beyond the whiskers indicate outliers. For the bar chart (B), the bar height indicates the mean, while the whiskers indicate the standard error.

**Figure S2.**


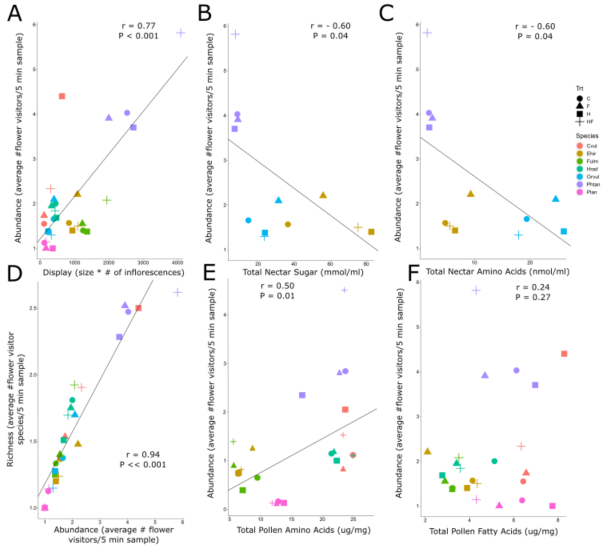


**Figure S2.** Correlations between flower-visitor abundance and floral display (A: average inflorescence size * number of inflorescences), total nectar sugar (B), total nectar amino acids (C), flower-visitor species richness (D), total pollen amino acids (E), and total pollen fatty acids (F) for the seven plant species and four experimental treatments. The Pearson Correlation Coefficient (r) and p value are provided for these correlations. The four experimental treatments are Control (C: circle), Fertilizer (F: triangle), Herbicide (H: square), and Combination (HF: cross). The plant species are *C. vulgare* (red), *E. hirsutum* (yellow), *F. ulmaria* (green), *H. radicata* (light blue), *O. vulgare* (blue), *P. tanacetifolia* (purple), *P. lanceolata* (pink).
